# The effect of continuous venovenous hemofiltration on neutrophil gelatinase-associated lipocalin plasma levels in patients with septic acute kidney injury

**DOI:** 10.1186/s12882-016-0363-y

**Published:** 2016-10-19

**Authors:** Xingui Dai, Tao Li, Zhenhua Zeng, Chunlai Fu, Shengbiao Wang, Yeping Cai, Zhongqing Chen

**Affiliations:** 1 Department of Critical Care Medicine, Nanfang Hospital, Southern Medical University, 1838 Guangzhou Avenue North, Guangzhou, Guangdong 510515 China; 2 Department of Critical Care Medicine, the First Peoples’ Hospital of Chenzhou, Institute of Translation Medicine, 102 Luo Jia Jin Street, Chenzhou, Hunan 423000 China

**Keywords:** Acute kidney injury, Continuous venovenous hemofiltration, Neutrophil gelatinase-associated lipocalin, Sepsis

## Abstract

**Background:**

It is known that continuous venonenous hemofiltration (CVVH) does not affect the plasma level of neutrophil gelatinase-associated lipocalin (pNGAL) in acute kidney injury (AKI) patients. However, because of the unique pathophysiology underlying AKI caused by sepsis, the effect of CVVH on pNGAL in this clinical setting is less certain. The purpose of this study was to determine the effect of CVVH on pNGAL in sepsis-induced AKI patients.

**Methods:**

Between August 1, 2014, and December 31, 2014, 42 patients with sepsis-induced AKI underwent CVVH in the general intensive care unit of our institution and were consecutively enrolled in this study. Prefilter, postfilter, and ultrafiltrate pNGAL measurements were taken at the initiation of continuous renal replacement therapy (CRRT) and repeated after 2, 4, 8, and 12 h (T0, T2h, T4h, T8h, and T12h, respectively). The mass transfer, plasma clearance, and sieving coefficient were calculated based on the mass conservation principle.

**Results:**

Following CVVH initiation, we found that pNGAL in the ultrafiltrate decreased significantly (*P* = 0.013); however, levels at the inlet and outlet showed no significant change (*P* > 0.05 for both). Furthermore, there was no change in the total mass removal rate, total mass adsorption rate, and plasma clearance over time (*P* > 0.05 for all), and a significant decrease in the sieving coefficient (*P* = 0.007) was seen.

**Conclusions:**

The results of this study show a limited effect of CVVH on pNGAL in sepsis-induced AKI patients. This suggests that pNGAL may be used as an indicator of renal progression in these patients. However, a larger study to confirm these findings is needed.

**Trial registration:**

ClinicalTrials.gov, NCT02536027. Retrospectively registered on 20th August 2015.

## Background

Acute kidney injury (AKI) is a leading cause of sepsis-related deaths in the intensive care unit (ICU), accounting for approximately half of all cases [1, 2]. Early diagnosis of AKI and timely prediction of renal recovery are two of the primary challenges in the field of AKI research. On one hand, because serum creatinine (SCr) does not accurately reflect the glomerular filtration rate (GFR), it is considered a late marker for AKI [3, 4]. On the other hand, the Beginning and Ending Supportive Therapy for the Kidney study suggested that a urine output >500 mL/d is the most important predictor of successful discontinuation of continuous venovenous hemofiltration (CVVH). However, the urinary output is often affected by clinical interventions (e.g., diuretic administration) [5]. Therefore, it is of great importance to discover a reliable biomarker for the early diagnosis of AKI that reflects the renal function of patients when they are receiving CVVH.

A growing body of evidence indicates that septic AKI is a different pathophysiological entity than other types of AKI. As such, the assessment and prognostic tools used to assess septic AKI need careful consideration, and this process has already started [6, 7]. In the last decade, a number of novel biomarkers to help diagnose AKI at an early stage and accurately predict renal recovery have been studied intensively. Plasma neutrophil gelatinase-associated lipocalin (pNGAL), a 25 kDa protein covalently bound to human neutrophil gelatinase, has been confirmed as a reliable biomarker for AKI occurrence and recovery from infectious disease [8–11]. However, patients requiring CVVH were excluded from most of these previous studies because it was thought that pNGAL concentration might change during CVVH through clearance as well as by release in the filter. A recent article by Schilder [12] reported that CVVH did not affect pNGAL in AKI patients due to low filter clearance. Another small series (*n* = 3) also reported that pNGAL was not significantly cleared during CVVH [13]. However, it remains unclear whether this also applies to septic AKI. The aim of the present study was to determine the effect of CVVH on NGAL concentrations in these patients.

## Methods

### Participants

The ethical committee of the First Peoples’ Hospital approved this study, and it was registered with the US National Institutes of Health Clinical Trials Register (NCT02536027). Patients or their next of kin signed informed consent forms of their own accord after being fully informed of all the relevant details of the study. All patients with septic acute kidney injury (SAKI) undergoing CVVH in the general ICU were consecutively enrolled in the study during the period from August 1, 2014, to December 31, 2015.

### Inclusion and exclusion criteria

Adult (>18 years) patients with SAKI undergoing continuous renal replacement therapy (CRRT) were assessed for inclusion. The urine output and SCr parameters were used from the 2012 Kidney Disease Improving Global Outcomes criteria [14], which were based on the Risk, Injury, Failure, Loss, End-stage/Acute Kidney Injury Network definitions. These included an absolute increase in SCr of ≥26.4 μmol/L over 48 h, a percentage increase in SCr of ≥50 % from baseline over the previous 7 days, or urine output ≤0.5 mL/(kg · h) for a period of ≥6 h. Furthermore, based on the diagnostic criteria of the 2001 International Sepsis Definition Conference [15], sepsis was defined as a systemic, deleterious host response to infection resulting in a systemic inflammatory response syndrome characterized by two or more of the following (definitions in parentheses): hypothermia or fever (body temperature <36 °C or >38.5 °C), tachycardia (>90 beats/min), tachypnea (>20 breaths/min or PaCO_2_ < 32 mm Hg on mechanical ventilation), leukocytosis (>12,000/mm^3^), leukopenia (<4000/mm^3^), or increased immature band forms (>10 %).

The following patients were excluded: (1) those with end-stage renal disease; (2) those with a history of renal transplant; (3) those with cancer; (4) those with acquired immunodeficiency syndrome; and (5) who had undergone high-dose steroid treatment.

### CRRT procedure

CVVH was performed with a Fresenius 4008S CRRT plus machine (Fresenius Medical Care, Homburg, Germany) after establishing venous access in the femoral or jugular vein with an 11- to 14-Fr double-lumen catheter. The hemodiafilter membrane used was Fresenius AV600S (Fresenius, Homburg, Germany). In principle, patients were hemofiltered at a blood flow rate of 180–220 mL/min in 2 L postdilution CVVH mode. An initial dose of heparin 400–1000 IU/h was given, with adjustment of the heparin infusion based on patient coagulation function. Anticoagulation also included a postfilter infusion of protamine, with a ratio of heparin 100 IU to protamine 1 mg.

### Data collection

Baseline patient data (age, gender, etiological factors, and underlying diseases) were collected on patient admission to the ICU. The white blood cell (WBC) count, C-reactive protein (CRP), and procalcitonin (PCT) levels were obtained at CVVH initiation. Clinical data necessary to calculate sequential organ failure assessment (SOFA) and acute physiology and chronic health evaluation II (APACHE II) scores were also collected.

### Measurement of plasma NGAL

Prefilter, postfilter, and ultrafiltrate samples were obtained at the beginning of CRRT and again after 2, 4, 8, and 12 h (T0, T2h, T4h, T8h, and T12h, respectively). The NGAL level was measured using an enzyme-linked immunosorbent assay (Lipocalin2/NGAL Duoset, DY1757, R&D Systems, UK) with a measurable range of 20–3000 ng/mL.

### Calculation

Based on the mass conservation principle, NGAL total mass removal rate (Mtr), mass adsorption rate (Mad), sieving coefficient (SC), and plasma clearance (PC) were calculated using the following formulas [12]:

$$ {Q}_{\mathrm{i}}={Q}_{\mathrm{b}}\times \kern0.5em \left(1\ \hbox{--}\ \mathrm{H}\mathrm{c}\mathrm{t}\right) $$

$$ {Q}_{\mathrm{o}}={Q}_{\mathrm{i}} $$

$$ {M}_{\mathrm{i}}={Q}_{\mathrm{i}}\times {C}_{\mathrm{i}} $$

$$ {M}_{\mathrm{o}}={Q}_{\mathrm{o}}\times {C}_{\mathrm{o}} $$

$$ {M}_{\mathrm{uf}}={Q}_{\mathrm{uf}}\times {C}_{\mathrm{uf}} $$

$$ {M}_{\mathrm{tr}}={M}_{\mathrm{i}}\hbox{--} {M}_{\mathrm{o}} $$

$$ {M}_{\mathrm{ad}}={M}_{\mathrm{tr}}\hbox{--} {M}_{\mathrm{uf}} $$

$$ \mathrm{P}\mathrm{C} = {M}_{\mathrm{tr}}/{C}_{\mathrm{i}} $$

$$ \mathrm{S}\mathrm{C} = 2 \times {C}_{\mathrm{uf}}/\left(C+{C}_{\mathrm{o}}\right) $$

Abbreviations:

*C*_i_: Concentration in inlet plasma before addition of replacement fluid (ng/mL)

*C*_o_: Concentration in outlet plasma (ng/mL)

*C*_uf_: Concentration in the ultrafiltrate (ng/mL)

*Q*_b_: Inlet blood flow rate (mL/min)

*Q*_i_: Inlet plasma flow rate (mL/min)

*Q*_o_: Outlet plasma flow rate (mL/min)

*Q*_uf_: Ultrafiltration flow rate (mL/min)

*M*_i_: Mass inlet rate (ng/min)

*M*_o_: Mass outlet rate (ng/min)

*M*_uf_: Mass ultrafiltration rate (ng/min)

*M*_tr_: Mass removal rate (ng/min)

*M*_ad_: Mass adsorption rate (ng/min)

RF: Replacement fluid flow rate (ng/min)

SC: Sieving coefficient

### Statistical analysis

Continuous variable data with normal distributions were provided as mean ± standard deviation (SD). Non-normally distributed continuous variable data were presented as median (25th, 75th percentiles). The Kruskal–Wallis test was used to compare measured and calculated data. Qualitative variable data were expressed as frequencies (*n*) and percentages (%). Statistical analyses were conducted using IBM SPSS 19.0 (SPSS; Chicago, IL, USA), and a *P* value of <0.05 was considered statistically significant.

## Results

Forty-eight consecutive subjects were screened. Of these, six subjects were excluded: three patients did not receive heparin anticoagulation due to severe coagulation disorders; one required an emergency operation due to active bleeding; one was excluded due to his next of kin withdrawing consent; and one patient died during CVVH, and his data were thus incomplete. Therefore, this study was completed for 42 patients, 24 males and 18 females, aged 53.2 ± 17.3 years. In 34 patients, venous access was established via the femoral vein, and in 8 patients, it was established via the jugular. Eighteen patients (42.9 %) died in the ICU. Nonsurvivors showed significantly higher median pNGAL at the inlet than survivors at T0 (1112 [323–1869] ng/mL versus 772 [121–1548] ng/mL, *P* = 0.033). Basic patient characteristics and clinical data at T0 are summarized in Tables 1 and 2, respectively.

**Table 1:** Clinical characteristics

Clinical characteristics	Patients with SAKI (*n* = 42)
Age (year)	53.2 ± 17.3
Male gender (*n*, %)	28 (66.7)
Etiological factors (*n*, %)	
Abdominal infection	18 (42.9)
Pulmonary infection	12 (28.6)
Trauma-related infection	10 (23.8)
Urinary tract infection	2 (4.8)
Underlying diseases (*n*, %)	
Hypertension	16 (38.1)
Diabetes	10 (23.8)
COPD	8 (19.0)
Coronary heart disease	6 (14.3)

Normally distributed quantitative data are presented as mean ± SD. Qualitative data are presented as *n* (%). *SAKI* septic acute kidney injury, *COPD* chronic obstructive pulmonary disease

**Table 2:** Biological data at T0

Biological data	Patients with SAKI (*n* = 42)
MAP (mm Hg)	67.2 ± 32.5
PaO_2_/FiO_2_	192 (131, 352)
SOFA	10 ± 3
APACHE II	18 (14, 21)
WBC count (×10^9^/L)	14.5 (11.3, 18.9)
Hct (%)	25.0 ± 5.3
PLT (×10^9^/L)	98 (46, 219)
CRP (mg/dL)	146.7 (53.3, 189.9)
PCT (ng/mL)	13.21 (3.02, 38.81)
SCr (μmol/L)	242 (189, 367)
BUN (mmol/L)	12.3 ± 8.4
Fibrinogen (g/L)	5.9 ± 3.2
Albumin (g/L)	28 ± 7.5
Lactate (mmol/L)	5.4 (2.2, 7.6)

Normally distributed quantitative data are presented as mean ± SD. Non-normally distributed quantitative data are presented as median (25th, 75th percentiles). Qualitative data are presented as *n* (%). *APACHE II* acute physiology and chronic health evaluation II, *BUN* blood urea nitrogen, *COPD* chronic obstructive pulmonary disease, *CRP* C-reactive protein, *Hct* hematocrit, *MAP* mean arterial pressure, *PCT* procalcitonin, *PLT* platelets, *SAKI* septic acute kidney injury, *SOFA* sequential organ failure assessment, *SCr* serum creatinine, *WBC* white blood cell

**Fig. 1:**
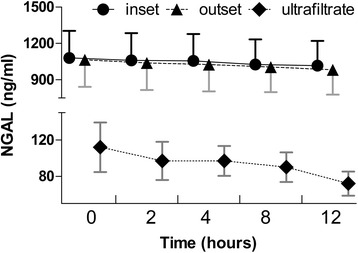
Plasma NGAL concentration over time at the inlet and outlet, and in the ultrafiltrate, after the initiation of CVVH in patients with SAKI. Levels of pNGAL at the inlet and outlet did not change (*P* > 0.05), whereas in the ultrafiltrate, the level decreased over time (*P* = 0.013). CVVH, continuous venovenous hemofiltration; NGAL, neutrophil gelatinase-associated lipocalin; SAKI, septic acute kidney injury

**Fig. 2:**
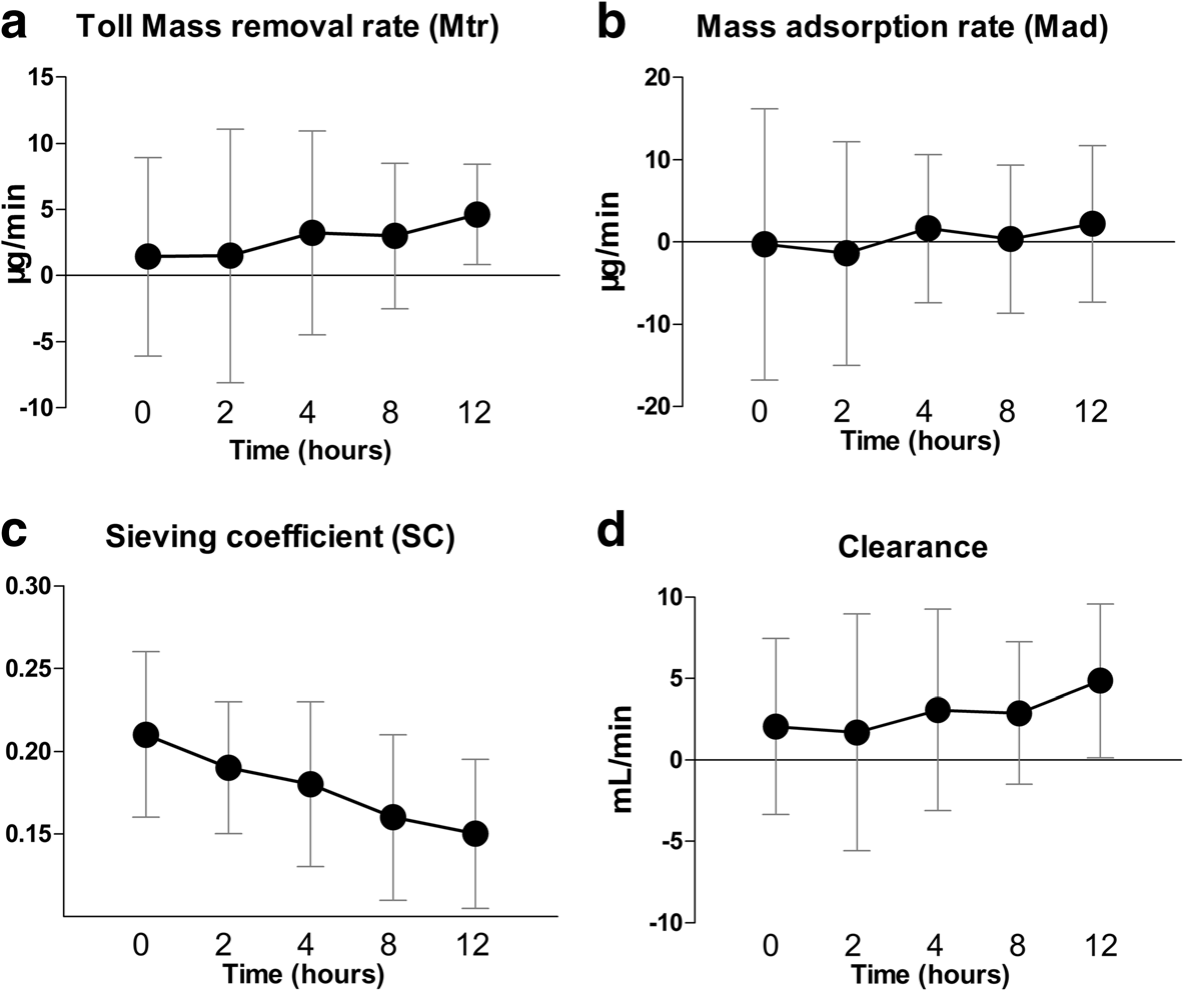
Total mass removal rate, mass adsorption rate, sieving coefficient, and clearance of pNGAL during CVVH. The total mass removal rate (**a**), mass adsorption rate (**b**), and plasma clearance (**d**) did not change over time, but the sieving coefficient (**c**) decreased significantly (*P* = 0.007). CVVH, continuous venovenous hemofiltration; NGAL, neutrophil gelatinase-associated lipocalin

All samples had detectable NGAL levels. Following initiation of CVVH, pNGAL in the ultrafiltrate decreased significantly (*P* = 0.013), whereas levels at the inlet and outlet did not change (*P* > 0.05 for both; Fig. 1). Furthermore, no change was seen in the total mass removal rate, total mass adsorption rate, and PC over time (*P* > 0.05 for all), while the SC significantly decreased (*P* = 0.007; Fig. 2). Table 3 presents data for the total mass removal rate, mass adsorption rate, SC, and PC of NGAL during CVVH.

**Table 3:** Total mass removal rate, Mass adsorption rate, Sieving coefficient, and Plasma clearance of NGAL during CVVH

	T0	T2	T4	T8	T12
*C*_i_ (ng/mL)	879 (733, 1466)	850 (685, 1525)	859 (688, 1496)	847 (689, 1417)	844 (694, 1405)
*C*_o_ (ng/mL)	867 (697, 1451)	845 (703, 1332)	890 (661, 1416)	834 (689, 1326)	819 (646, 1415)
*C*_uf_ (ng/mL)	32.2 (26.7, 40.8)	30.3 (23.6, 34.9)	27.7 (24.1, 31.9)	26.1 (21.2, 30.1)	24.0 (20.1, 28.0)
*M*_i_ (μg/min)	107 (94, 183)	104 (97, 194)	105 (89, 197)	105 (91, 182)	110 (88, 180)
*M*_o_ (μg/min)	104 (92, 181)	107 (88, 170)	109 (85, 182)	107 (89, 170)	109 (85, 172)
*M*_uf_ (ng/min)	2189 (1330, 2748)	1807 (1181, 3363)	1888 (1309, 2312)	2048 (1129, 3693)	1397 (1018, 3471)
*M*_tr_ (ng/min)	1430 (239, 4914)	1502 (−2500, 4801)	3240 (593, 5544)	3024 (−82, 5360)	4620 (2540, 6687)
*M*_ad_ (ng/min)	−313(−2576, 3470)	−1369 (−5611, −1682)	1651 (−1494, 3308)	354 (−2195, 3201)	2216 (507, 5104)
SC	0.21 (0.17, 0.26)	0.19 (0.16, 0.22)	0.17 (0.15, 0.21)	0.16 (0.13, 0.20)	0.16 (0.12, 0.18)
Clearance	2.05 (0.39, 3.57)	1.45 (−3.15, 4.28)	3.74 (0.73, 6.09)	3.56 (−0.45, 5.80)	4.57 (1.85, 8.94)

Data are presented as median (25th, 75th percentiles). *CVVH* continuous venovenous hemofiltration, *NGAL* neutrophil gelatinase-associated lipocalin, *C*_i_ concentration in inlet plasma before addition of replacement fluid, *C*_o_ concentration in outlet plasma, *C*_uf_ concentration in the ultrafiltrate, *M*_ad_ mass adsorption rate, *M*_uf_ mass ultrafiltration rate, *M*_i_ mass inlet rate, *M*_o_ mass outlet rate, *M*_tr_ mass removal rate, *SC* sieving coefficient

## Discussion

Results of this study demonstrate that CVVH did not significantly affect the plasma concentration of NGAL in patients with septic AKI: pNGAL did not decline, and NGAL clearance was lower than expected. There is considerable evidence that the underlying pathophysiology of septic AKI is unique in comparison with those of other types of AKI. Specifically, NGAL levels differ significantly between AKI patients with and without sepsis [16]. Therefore, it is reasonable to surmise that the clearance and production of NGAL during CVVH may be different than in other types of AKI. However, this study yielded results in accordance with the previous studies on AKI from other causes [12, 13]. A three-case study demonstrated that CVVH did not substantially affect pNGAL concentration in AKI patients [12]. Another study by Schilder and colleagues [13] also reported no net removal of NGAL during CVVH in patients with AKI. These findings indicate that CVVH does not affect the levels of NGAL in AKI patients with or without sepsis.

Contrary to the present study results, however, Donadio recently suggested that dialytic techniques and membranes can remove pNGAL and affect its accuracy as a biomarker of AKI [17]. They reported that low-flux dialysis (F8; Fresenius, Bad Homburg, Germany) did not remove pNGAL (which increased by 9.1 ± 24.4 %), whereas high-flux dialysis (N190 FH; Nipro, Osaka, Japan; triacetate cellulose membrane, surface area 1.9 m^2^; ultrafiltration rate 8474 mL/h per 100 mm Hg) decreased pNGAL significantly (*P* < 0.0001). What is more, they found that the reduction ratio of pNGAL with hemodiafiltration using a polyphenylene membrane (surface area 2.0 m^2^) and an ultrafiltration rate of 6800 mL/h per 100 mm Hg (Phylther; Bellco, Mirandola, Italy) or an acrylonitril and natrium metallylsulfone copolymer membrane (surface area 2.15 m^2^) and an ultrafiltration rate of 6500 mL/h per 100 mm Hg (Nephral 500; Gambro, Lund, Sweden) were higher than those seen with high-flux dialysis (52.1 ± 26.7 % vs 26.6 ± 26.1 %, *P* = 0.053). In the present study, CVVH treatments were performed using a polysulfone membrane (AV600s; Fresenius, Homburg, Germany; surface area of 1.35 m^2^), with a cutoff of approximately 30 kDa and an ultrafiltration rate of 4000 mL/h per 100 mm Hg. It is conceivable that membrane characteristics may impact NGAL removal significantly. In clinical practice, in view of different membrane characteristics among different CRRT machines, the result should be discriminatory. For example, the efficacy of NGAL removal using a AN69 membrane (M100 set; Prismaflex, Hechingen, Germany) with a surface area of 0.9 m^2^ may be lower than that using a high-flux membrane. Therefore, further studies are needed to confirm whether the membrane type as well as the dialytic techniques used affect the clearance of NGAL in patients with septic AKI.

The present study had certain limitations. First, it was a single-center observational study with a small cohort of patients, which increased the chance of a type II error. A larger study is required to confirm the present finding. Second, the impact of anticoagulation was not considered. Schilder [12] reported that pNGAL was not affected by the anticoagulation of patients with AKI. However, another study demonstrated that sepsis was frequently associated with the pathological activation of the coagulation system [18]. Thus, it remains unclear whether or not pNGAL is affected by anticoagulation in patients with septic AKI receiving CVVH.

## Conclusions

This study confirmed that pNGAL does not significantly decrease during CVVH in septic AKI. This means that the impact of CVVH does not need to be considered when pNGAL is used to judge renal progression in patients with septic AKI who are receiving CVVH.

## Abbreviations

AKI: Acute kidney injury
APACHE II: Acute physiology and chronic health evaluation II
CRRT: Continuous renal replacement therapy
CVVH: Continuous venovenous hemofiltration
ICU: Intensive care unit
NGAL: Neutrophil gelatinase-associated lipocalin
SOFA: Sequential organ failure assessment
UFR: Ultrafiltration rate
